# ImmunoResponse Predictor: a GUI for accurate response prediction to immunotherapy

**DOI:** 10.7717/peerj.21553

**Published:** 2026-08-06

**Authors:** Rajat Butola, Shinsheng Yuan, Grace S. Shieh

**Affiliations:** 1 Institute of Statistical Science, Academia Sinica, Taipei, Taiwan; 2 Bioinformatics Program, Taiwan International Graduate Program, Academia Sinica, Taipei, Taiwan; 3 Genome and Systems Biology Degree Program, Academia Sinica, National Taiwan University, Taipei, Taiwan; 4 Data Science Degree Program, Academia Sinica and National Taiwan University, Taipei, Taiwan

**Keywords:** Biomarker, Immunotherapy, Cancer, Machine learning, GUI, Regression, Prediction

## Abstract

Immune checkpoint inhibitors (ICIs) have improved outcomes for subsets of patients with metastatic urothelial carcinoma (mUC) and metastatic renal cell carcinoma (mRCC), yet objective response rates remain low (∼15–25%), underscoring the need for tools that support patient stratification. We previously developed LogitDA, a logistic regression–based predictor incorporating feature selection and domain adaptation, which outperformed established immune-related signatures in predicting response to the PD-L1 inhibitor atezolizumab. Here, we present the ImmunoResponse Predictor, a web-based clinical decision-support framework that enables responsible application of LogitDA in real-world settings. The system integrates standardized transcriptomic preprocessing, interpretable individual-level predictions (including single-sample use), clinically motivated LogitDA score cutoffs that prioritize minimization of false negatives, and a cohort-level percentage of applicability with empirically derived thresholds designed to assess whether predictions can be reliably extrapolated to newly uploaded datasets. Importantly, applicability functions as a diagnostic safeguard against distributional shift rather than as a response predictor. We evaluated the framework across four independent cohorts, PCD4989g(mUC), PCD4989g(mRCC), the Moreno cohort, and the UNC-108 cohort. LogitDA achieved prediction accuracies of 0.69, 0.83, 0.86, and 0.53, with corresponding applicability estimates of 74%, 76%, 71%, and 48%, respectively, correctly identifying the UNC-108 cohort as one in which predictions warrant increased caution. Overall, the ImmunoResponse Predictor extends LogitDA into a practical, interpretable, and safeguarded tool for immunotherapy response prediction, supporting cautious clinical and translational use.

## Introduction

Urothelial carcinoma (UC) is the ninth most common cancer worldwide, while renal cell carcinoma (RCC) accounts for approximately 90% of all kidney cancers. In 2022, an estimated 552,000 and 413,000 new cases of UC and RCC were reported globally (World Cancer Research Fund, 2025). Metastatic urothelial carcinoma (mUC) and metastatic renal cell carcinoma (mRCC) are highly aggressive malignancies, accounting for ∼90% of deaths from UC and RCC. In recent years, immunotherapies, in particular immune checkpoint inhibitors (ICIs), have reshaped the treatment landscapes for mUC and mRCC (Gajate et al., 2020). These agents target immune checkpoints such as PD-1 and PD-L1, which play important roles in human immune system. Specifically, Atezolizumab, this humanized monoclonal antibody selectively blocks PD-L1 from interacting with PD-1 and the co-stimulatory molecule B7.1. By disrupting these immunosuppressive interactions, atezolizumab restores tumor-specific T-cell activity and enables a robust immune response.

In patients who respond to ICIs, *e.g.*, atezolizumab, overall survival (OS) can be significantly prolonged. As a result, ICIs have become frontline treatments in selected cases and the standard of care for second-line therapy following progression on platinum-based chemotherapy (Powles et al., 2018). However, the objective response rate (ORR) remains low-approximately 15–25% in mUC (Powles et al., 2018) and mRCC (Motzer et al., 2015). This underscores the critical, yet challenging, need to identify potential responders prior to treatments (Havel, Chowell & Chan, 2019; Ma et al., 2021).

Using machine learning and innovative feature selection techniques (Wang et al., 2022), including unsupervised domain adaptation (Jeng et al., 2023), we previously developed predictors for cancer patient responses to chemo-, targeted-, and immunotherapies (Yuan et al., 2023; Langfelder et al., 2025a). Notably, a logistic regression-based predictor (LogitDA) demonstrated the ability to generalize response prediction to atezolizumab (a PD-L1 inhibitor) from training data to independent test datasets (Langfelder et al., 2025b). LogitDA identified a 49-gene signature for metastatic urothelial carcinoma (mUC) and a 27-gene signature for metastatic renal cell carcinoma (mRCC); see Table S1 for the signature genes. With these signatures, LogitDA achieved an area under the curve (AUC) of 75% for mUC and 72% for mRCC. Moreover, LogitDA outperformed six established signatures, including PD-L1 IHC (Banchereau et al., 2021), the IFN-γ signature, the CD8T signature, the T-cell dysfunction signature, the T-exhaust signature, and the T-inflamed signature (Ayers et al., 2017; Lakatos et al., 2020; Mariathasan et al., 2018; Jiang et al., 2018; Cristescu et al., 2018).

In this study, we developed *ImmunoResponse Predictor*, a web-based graphical user interface (GUI). This GUI is based on LogitDA and its associated gene signatures for mUC and mRCC, which can predict patient response (including single samples) to immunotherapy. We also trained LogitDA score cutoffs that prioritize minimizing false negatives of predictions, and derived an applicability metric with empirically derived thresholds to assess whether LogitDA predictions can be reliably extrapolated to new cohorts. This GUI is demonstrated using four independent cohorts in mUC and mRCC. Overall, this user-friendly platform is designed to support clinicians and researchers by delivering ICI response predictions together with explicit safeguards for their interpretation in advanced or metastatic UC and RCC.

## Materials & Methods

### Training datasets

We used the IMvigor210 and the IMmotion150 cohorts as the training datasets, which were collected as previously described in Langfelder et al. (2025b).

### Independent test datasets

We used PCD4989g (NCT01375842; Herbst et al., 2014) as test datasets in mUC and mRCC, which were obtained as previously described in Langfelder et al. (2025b). Additionally, we collected whole-transcriptome profiles of 89 and 11 patients with mUC (Rose et al., 2021; GSE111636 (henceforth the Moreno cohort)), which served as unseen cohorts to demonstrate how this GUI can be used to guide treatment in clinics. Clinical responses in the IMvigor210, PCD4989g, and IMmotion 150 datasets were assessed according to RECIST 1.1 (Schwartz et al., 2016), and were grouped into responders (CR/PR) and non-responders (SD/PD), respectively, as described in Langfelder et al. (2025b). In total, the IMvigor210 (IMmotion150) cohort included 298 (77) patients, with 68 (15) responders and 230 (62) non-responders. The PCD4989g test dataset for mUC (mRCC) included 94 (58) patients, with 22 (eight) responders and 72 (50) non-responders. Similarly, the clinical response in the UNC-108 and Moreno cohorts were classified by RECIST 1.1, of which there are 16 (∼18%) and six (∼55%) responders out of 89 and 11 samples in total. IMvigor210 dataset is available in European Genome-Phenome Archive (EGA) under accession number EGAS00001002556. Data for the clinical trials IMmotion150 and PCD4989g are available under restricted access in European Genome-Phenome Archive with the reference number EGAS00001004343. Raw and clinical data of PCD4989g were accessed *via* Genentech’s internal Strand pipeline (South San Francisco, USA), and downloaded using pyEGA3 (https://github.com/EGA-archive/ega-download-client). Both the UNC-108 and Moreno cohorts were downloaded from GEO with accession number GSE176307 and GSE111636, respectively.

### Standardization of training and test data

We implemented the same standardization procedures for training and test data as described in the Methods of Langfelder et al. (2025b).

### Clinically motivated performance metrics and the trained cutoffs

Let *D*_*train*_ denote the training dataset, which was selected such that its sample size and event rate were close to the observed objective response rate. This was intended to ensure that *D*_*train*_ is representative of the target population with respect to response prevalence. Let s_i_ denote the LogitDA score for subject *i*, and let y_i_ ∈ {0,1} denote the observed response status, where 1 and 0 correspond to responder and non-responder, respectively.

To reflect the clinical priority of minimizing false negatives (more important than false positives), we adopted an asymmetric decision thresholds rather than the default probability cutoff of 0.5. Specifically, we define an upper cutoff *c*_*U*_for responder prediction and a lower cutoff *c*_*L*_ for non-responder prediction, where ${c}_{U}\in \left[ 0.6,0.9 \right] $ and *c*_*L*_ ∈ [0.1, 0.4], each evaluated in increments of 0.01.

For each candidate cutoff pair (*c*_*U*_, *c*_*L*_), predictions are defined as follows.

\begin{eqnarray*}{\hat {y}}_{i}= \left\{ \begin{array}{@{}l@{}} \displaystyle 1, {s}_{i}> {c}_{U}, \\ \displaystyle 0, {s}_{i}< {c}_{L}, \\ \displaystyle \mathrm{NA}, \mathrm{otherwise}.  \end{array} \right. \end{eqnarray*}

Using the IMvigor210 cohort as the training set, we evaluated each candidate cutoff pair according to three criteria, ranked lexicographically: (1) minimum false negative rate, (2) maximum true positive rate, and (3) maximum precision.

The top five cutoff pairs were retained, and the top-ranked pair was selected. In our implementation, this resulted in an upper cutoff *c*_*U*_ = 0.50, and a lower cutoff *c*_*L*_ = 0.29. These LogitDA score thresholds were then fixed and applied to the four independent datasets, *e.g.*, PCD (mUC), and future datasets. Accordingly, for any new subject, s_i_ > 0.50 is predicted as responder, s_i_ < 0.29 is predicted as non-responder, and intermediate scores are classified as not applicable (*i.e.,* NA).

Because the fixed score cutoffs are sensitive to scale, this procedure assumes that future data are on the same scale as the training data. Therefore, we recommend applying the trained cutoffs only to properly normalized data. To support this requirement, we have incorporated a machine learning-based normalization procedure into the GUI implementation.

### The cosine distance

The cosine distance was used to quantify similarity between an uploaded sample and responder/non-responder groups in the training cohort based on gene-expression profiles. The cosine distance between an uploaded sample **x**_*K*_ and the Rs (NRs) of the test set **y**_**1j**_ (**y**_**2j**_**)** is defined as follows.

\begin{eqnarray*}\text{Cosine distance}=1- \frac{{\mathop{\sum \nolimits }\nolimits }_{j=1}^{{J}_{i}}{\mathop{\sum \nolimits }\nolimits }_{k=1}^{K}{\mathrm{x}}_{\mathrm{k}}{\mathrm{y}}_{\mathrm{ijk}}}{\sqrt{{\mathop{\sum \nolimits }\nolimits }_{k=1}^{K}{x}_{k}^{2}}\sqrt{{\mathop{\sum \nolimits }\nolimits }_{k=1}^{K}{y}_{ijk}^{2}}} , \end{eqnarray*}

where *i* = 1 and 2, and **y**_**1j**_ (**y**_**2j**_**)** denote the *j*th responder R (non-responder NR) in the test data used for domain adaptation in training LogitDA, y_ijk_ and x_k_ represent the kth gene of the signature in the training sample y_ij_ and a new uploaded data. For example, *K* = 49 for mUC, **y**_**1**_ contains 68 Rs and **y**_**2**_ contains 230 NRs in the IMvigor210 training set.

While cosine distance provides an intuitive measure of proximity, its direct use for classification requires ad hoc thresholds that are difficult to justify clinically. Furthermore, in a pilot study the cosine distance method yielded only 70% applicability in PCD4989g(mUC) and 60% in PCD4989g(mRCC), which did not reflect their prediction accuracy of 75% and 83% using the default cutoff 0.50 for LogitDA scores (Tables S2A and S2B), respectively.

### ORR information-based cosine distance and the applicability metric

To address the limitations of cosine distance, we developed an ORR-informed cosine distance metric (iCosineDist) method and the associated percentage of applicability. Based on large clinical studies reporting ORRs of approximately 15–25% for patients with mUC and mRCC treated with ICIs (Powles et al., 2018; Motzer et al., 2015), we adopted a median ORR of 20% as the expected responder proportion. For a newly uploaded cohort, samples were ranked by their average cosine distance to the responder group, and the top 20% were labeled as responders, with the remainders labeled as NRs, thereby defining the iCosineDist labels.

We define the percentage of applicability, a metric that quantifies the applicability of LogitDA to unseen datasets, as follows.

\begin{eqnarray*}\text{Percentage of applicability}= \left( \frac{\text{total matches}}{\text{total samples}} \right) \times 100\%, \end{eqnarray*}

where *total matches* denotes the number of samples whose LogitDA-predicted labels are consistent with those assigned by iCosineDist, and *total samples* denotes the total number of uploaded test samples.

### Empirical thresholds for applicability of LogitDA to new cohorts

Using the IMvigor210 cohort as the training reference, we empirically examined the relationship between cohort-level applicability and prediction performance across three independent mUC and one mRCC cohorts. We observed that cohorts with applicability above approximately 70% consistently retained stable prediction performance (the PCD4989g(mUC and mRCC), Moreno and cohorts), whereas cohorts with the percentage of applicability below approximately 60% showed marked performance degradation (*e.g.*, the UNC-108 cohort), indicating a distributional shift relative to the training data. Based on these observations, we adopted 70% and 60% as practical upper and lower bounds, respectively, to guide interpretation of model outputs. These thresholds are empirically observed heuristics rather than formal decision rules.

## Results

### The architecture of ImmunoResponse Predictor

This section outlines the structure of ImmunoResponse Predictor (the GUI), which comprises two main components: the *Interface Unit* and the *Computing Unit*. The overall workflow is depicted in Fig. S1, illustrating the integration between user interaction and backend computation.

(a) **Interface unit:** The Interface Unit serves as the front end of the system, where users can upload a gene-expression matrix (log_2_ (TPM+1)) and select the appropriate pre-trained LogitDA model (mUC or mRCC). The ImmunoResponse Predictor accepts a pre-treatment RNA-seq count or microarray matrix in .csv format, where rows represent samples and columns represent genes. The column names comprise either the 49-gene mUC signature and the 27-gene mRCC signature, or whole-transcriptome data for either cancer type, derived derived from biopsy or surgical samples collected prior to initiation of immunotherapy. The tool can accept four types of gene identifiers (gene symbol, Entrez gene IDs, Ensembl gene ID and Ensembl transcript ID). If some signature genes are absent, the system reports an error and prompts correction before prediction. Once a file is submitted, the interface passes the data to the Computing Unit, and the GUI predicts each sample as a responder (R), not applicable (NA), or a non-responder (NR), respectively. Finally, the GUI outputs a .csv file containing the sample IDs, LogitDA scores, predicted labels, and the percentage of applicability for a cohort.
(b) **Computing unit:** This backend module first extracts the expression values of the signature genes of LogitDA from the uploaded test data for mUC (mRCC), respectively. The GUI preprocesses the uploaded data by converting multiple gene IDs into Entrez IDs, conducting batch correction, and applying quantile normalization for mRCC cohort. Next, the system predicts each sample as either a R, not applicable (NA), or NR, and computes the percentage of applicability that quantifies how well LogitDA applies to new cohorts (see Methods).

### The GUI usage

The GUI reports sample-level LogitDA scores with predicted labels and a cohort-level applicability score, provided multiple samples are uploaded.

### Demonstration using real datasets

In this section, we first demonstrate the efficacy of the ImmunoResponse Predictor in predicting responses to immune checkpoint inhibitor (ICI) treatments using the PCD4989g cohorts (NCT01375842; Banchereau et al., 2021). These independent test datasets comprise whole-transcriptome RNA-seq profiles and corresponding clinical information from 94 and 58 patients with metastatic urothelial carcinoma (mUC) and metastatic renal cell carcinoma (mRCC), respectively. Hereafter, these cohorts are referred to as PCD(mUC) and PCD(mRCC).

**Figure 1:**
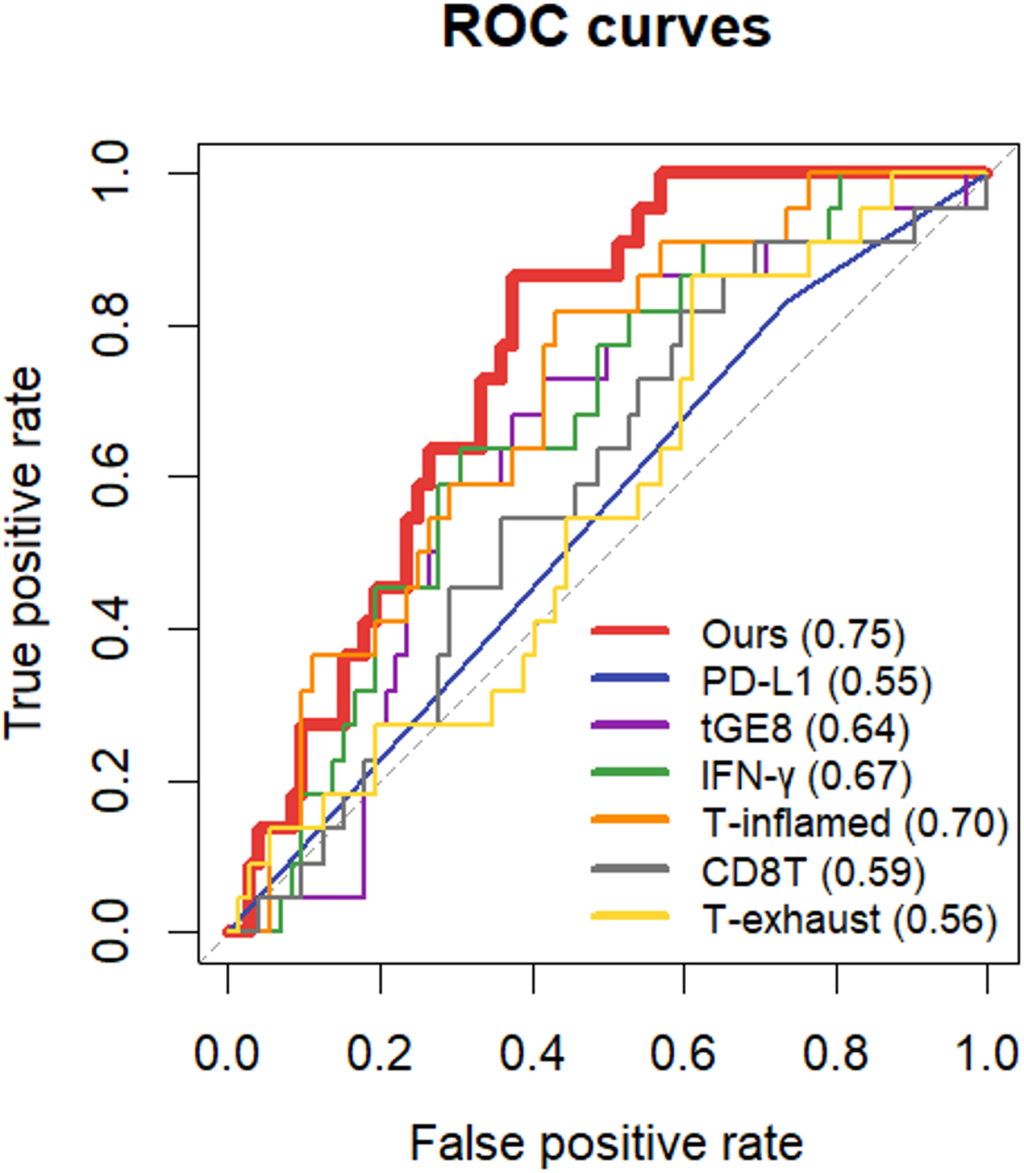
The prediction AUC of the ROC curve of LogitDA with our signature (ours) and the six existing signatures (listed in Table S1).

With feature selection procedures that include unsupervised domain adaptation, LogitDA demonstrated strong predictive performance, as reflected by high AUC values across independent datasets (Langfelder et al., 2025a). In the PCD(mUC) cohort, LogitDA achieved a prediction AUC of 0.75 with an accuracy of 71%, while in mRCC it reached an AUC of 0.72 with an accuracy of 83%. Figure 1 illustrates the predictive performance of LogitDA, as measured by area under the ROC curve (AUC), applied to the PCD(mUC) cohort. The prediction results for LogitDA with all evaluated signatures are provided in Tables S2A and S2B, in which LogitDA with the identified mUC (49 genes) and mRCC (27 genes) signatures outperformed several existing immune-related signatures (Langfelder et al., 2025a). Collectively, these findings underscore the strong predictive capability of LogitDA with ours for immunotherapy response and its potential to improve patient stratification in mUC and mRCC.

### Demonstration of LogitDA with the trained cutoffs using four independent cohorts

In this study, we additionally collected two mUC cohorts from the public domain (Rose et al., 2021; GSE111636; see Materials), hereafter referred to as UNC-108 and Moreno, to evaluate performance of LogitDA on newly uploaded datasets. From a clinical perspective, avoiding false negatives (FNs), patients who could benefit from immune checkpoint inhibitors (ICIs) but are incorrectly predicted as non-responders, is generally more critical than minimizing false positives (FPs), particularly in mUC and mRCC where durable responders exist. Accordingly, we used the IMvigor210 cohort to train LogitDA score cutoffs to reflect this clinical priority (see Methods).

Test samples from the PCD(mUC), PCD(mRCC), Moreno, and UNC-108 cohorts were classified as responder (R), NA, or non-responder (NR) according to the trained cutoffs (see Methods). LogitDA with our signature (ours) demonstrated stable performance in the PCD(mUC), PCD(mRCC), and Moreno cohorts, but only moderate performance in the UNC-108 cohort. The corresponding prediction accuracies of 69%, 83%, 86%, and 53%, respectively. The associated false negative rates (FNRs; corresponding precisions) were 0% (41%) for PCD (mUC), 33% (40%) for PCD (mRCC), 25% (100%) for Moreno, and 27% (24%) for UNC-108 (see Table 1 for details).

**Table 1:** Performance of LogitDA with four signatures in response prediction to ICIs across four cohorts.

**A. The PCD4989g(mUC) cohort**										
Signatures	CV result	Prediction result^a^								
	AUC (SE) (%)	AUC (%)	accuracy (%)	FNR (%)	Recall (%)	Precision (%)	TPs	TNs	FPs	FNs
Ours^b^	77	75	69	0	100	40	12	25	17	0
PD-L1	57	55	40	17	83	26	15	15	42	3
tGE8 (8)	68	65	61	45	55	31	12	45	27	10
IFN-γ (18)	70	67	67	41	59	37	13	50	22	9

**B. The PCD4989g(mRCC) cohort**										
Ours	95	72	83	33	67	40	4	34	6	2
PD-L1	57	53	38	25	75	15	6	15	33	2
tGE8 (8)	60	65	50	38	63	16	5	24	26	3
IFN-γ (18)	66	56	59	63	38	14	3	31	19	5

**C. The Moreno cohort**										
Ours	77	87	86	25	75	100	3	3	0	1
PD-L1	55	28	36	83	17	33	1	3	2	5
tGE8 (8)	68	53	45	66	33	50	2	3	2	4
IFN-γ (18)	70	50	64	50	50	75	3	4	1	3

**D. The UNC-108 cohort**										
Ours	77	61	53	27	73	24	8	24	26	3
PD-L1	55	62	64	38	63	28	10	47	26	6
tGE8 (8)	68	59	55	50	50	20	8	41	32	8
IFN-γ (18)	70	68	66	44	56	28	9	50	23	7

**Notes.**

a The prediction results of LogitDA with ours were obtained using the trained cutoffs for LogitDA scores.

b Ours denotes the identified gene signatures of LogitDA for mUC/mRCC (Langfelder et al., 2025a; Langfelder et al., 2025b), the genes of tGE8 and IFN-γ signatures are listed in Table S1.

Next, we compared LogtDA with ours to LogitDA using three well-established signatures, PD-L1 (Banchereau et al., 2021), tGE8 (an eight-gene T cell–related signature; Lakatos et al., 2020), and IFN-γ (Ayers et al., 2017). As shown in Table 1, LogitDA with our signature outperformed LogitDA with these signatures across the PCD(mUC), Moreno, and UNC-108 cohorts in terms of lower false negative rates and higher recall, ranking second only to PD-L1. However, our approach substantially outperformed PD-L1 in precision (40% *vs.* 15%), as PD-L1 identified only six true positives among 39 predicted positives.

### Clinical use and interpretation of outputs

We further examined the relationship between cohort-level applicability (see Methods) and prediction performance of LogitDA across these independent datasets. As shown in Table 2, cohorts with applicability values above approximately 70%, including the PCD(mUC), PCD(mRCC), and Moreno cohorts, consistently retained stable prediction performance. In contrast, the UNC-108 cohort exhibited applicability below 60% and achieved a prediction accuracy of only 53%, indicating a substantial shift relative to the training data. Notably, applicability values computed using CosineDist for the PCD(mRCC) and UNC-108 cohorts (60% and 73%, respectively) were inconsistent with their corresponding prediction accuracies (83% and 53%). By comparison, applicability based on iCosineDist more closely reflected observed performance. Accordingly, we recommend using iCosineDist-based applicability for future uploaded samples (see Table 2 for details).

**Table 2:** Prediction performances of LogitDA versus cohort applicability values across four independent cohorts.

**Dataset**	**Sample** **size**	**Responder**	**Non-Responder**	**Percentage of responders (%)**	**Accuracy** **(%) the** **trained cutoffs** ^a^	**% of Applicability (iCosineDist** ^b^ **)**	**% of** **Applicability (CosineDist)**
PCD(mUC)	94	22	72	23	69	74	70
PCD(mRCC)	58	8	50	14	83	76	60
Moreno	11	6	5	55	86	71	73
UNC-108	89	16	73	18	53	48	73

**Notes.**

a Predictions were based on the trained cutoffs: LogitDA scores >0.50 were classified as responders, scores <0.29 as non-responders, and intermediate scores (0.29 <score ≤ 0.50) as not applicable (NA). NA samples were excluded from performance calculations.

b The iCosineDist and CosineDist denote the iCosine distance and cosine distance, respectively.

Based on these observations, we interpreted applicability values above 70% as indicating reliable predictions, values below 60% as unreliable, and values between 60% and 70% as requiring cautious interpretation. Importantly, these thresholds are not intended to serve as rigid clinical decision rules, but rather as conservative safeguards to guide interpretation of model outputs.

Details of cutoff derivation and applicability computation are provided in the Methods.

Table 3 summarizes the outputs of the ImmunoResponse Predictor for the Moreno cohort, including LogitDA scores, predicted labels based on the trained cutoffs, iCosineDist-based labels, and the corresponding applicability values. Complete prediction results for the PCD(mUC), PCD(mRCC), Moreno, and UNC-108 cohorts are provided in Table S3.

**Table 3:** The output of ImmunoResponse prediction, including percentage of applicability, of the Moreno Cohart.

**SampleID**	**CosDist_2_Rs**	**CosDist_2_NRs**	**LogitDA_Score**	**LogitDA_** **score_label** ^a^	**True_label** ^a^	**iCosinDist_** **label**	**% of applicability**
GSM3036125	0.81	1.07	0.55	R	R	R	71
GSM3036135	0.90	1.03	0.32	NA	NR	R	
GSM3036129	0.91	1.03	0.92	R	R	NR	
GSM3036133	0.93	1.03	0.30	NA	NR	NR	
GSM3036127	0.96	1.03	0.54	R	R	NR	
GSM3036134	0.96	1.02	0.26	NR	NR	NR	
GSM3036132	1.06	0.98	0.27	NR	NR	NR	
GSM3036130	1.09	0.98	0.24	NR	R	NR	
GSM3036131	1.10	0.97	0.24	NR	NR	NR	
GSM3036128	1.11	0.95	0.44	NA	R	NR	
GSM3036126	1.14	0.94	0.33	NA	R	NR	

**Notes.**

a LogitDA_ score_label, True_label^a^ and iCosinDist_label denote the labels (R, NA or NR) assigned based on LogitDA scores, the observed responses in the Moreno cohort, and the iCosineDist metric, respectively.

Cohort-level applicability is critical for interpretation. When the percentage of applicability is high (*e.g.*, above 75%), outputs from the GUI may be used to support clinical judgement. Conversely, even when predicted labels are strong (*i.e.,* associated with high LogitDA scores), results should be interpreted cautiously if applicability is low. In particular, applicability values below 60% indicate limited similarity between the uploaded data and the training cohorts, suggesting that the GUI may not be applicable to these data. The cohort-level percentage of applicability is not intended to replace individual predictions. For very small sample sizes, this metric is not required for generating predictions and should be interpreted cautiously; when applicable, it provides complementary information on model reliability rather than a prerequisite for individual-level clinical use. Moreover, although transcriptomic predictors capture key aspects of tumor-immune biology, they do not account for all host-, treatment-, or disease-related factors that influence clinical outcomes. Accordingly, the ImmunoResponse Predictor is intended to support, rather than replace, clinical judgment.

### Single-sample prediction and output interpretation

Because a common clinical use case involves evaluating a single patient or a small number of patients, the ImmunoResponse Predictor provides individual-level predictions directly for single samples (Table S4). The percentage of applicability is not required to generate individual-level predictions and is therefore not reported for single-sample inputs. For very small sample sizes, this metric should be interpreted cautiously, as it primarily reflects cohort-level model reliability rather than individual-level prediction confidence.

The trained LogitDA score cutoffs (>0.50 for responders and <0.29 for non-responders) remain applicable for single-sample predictions. As a practical rule of thumb when interpreting GUI outputs for individual samples, LogitDA scores closer to 1.0 indicate a higher likelihood of response to immune checkpoint inhibitors (ICIs), whereas scores closer to 0 indicate a higher likelihood of non-response; scores near the decision cutoffs should be interpreted with caution.

### Other clinical factors to be considered when interpreting the outputs

Predictions generated by the ImmunoResponse Predictor should be interpreted within the broader clinical context, including prior treatments (*e.g.*, chemotherapy or targeted therapies that may alter the tumor immune microenvironment and gene expression profiles), line of therapy (as response rates and durability are generally higher in earlier treatment lines), patient characteristics such as age, sex, and performance status (*e.g.*, ECOG), and tumor features including histology and molecular subtype.

### Installation and dependencies

Users can access the ImmunoResponse Predictor at GitHub, and it can be downloaded for local use from https://github.com/gshieh/ImmunoResponsePredictor. The application is implemented in R (v4.3.2; R Core Team, 2023) using the Shiny framework for both preprocessing and prediction, leveraging R’s robust ecosystem. The system runs on a Windows platform powered by an Intel^®^ Core™ i7-13700 processor with 16 GB of RAM.

## Discussion

The ImmunoResponse Predictor was developed to facilitate practical use of transcriptomic response models while explicitly addressing challenges associated with external validation. Built on LogitDA and identified gene signatures for mUC and mRCC, the GUI enables non-technical users to generate response predictions to PD-L1 inhibitors such as atezolizumab with minimal technical burden. The tool is intentionally framed as a decision-support system: individual-level predictions are provided for single patients, while cohort-level applicability functions as a conservative safeguard when uploaded data deviate from the validated domain.

A key limitation of transcriptomic predictors is their sensitivity to distributional shift, which can compromise reliability when applied to unseen cohorts. To mitigate this risk, the GUI integrates clinically motivated LogitDA score cutoffs together with an explicit assessment of cohort-level applicability, allowing users to identify settings in which predictions should be interpreted cautiously. Cutoffs were derived by prioritizing clinically relevant error trade-offs, specifically minimizing false negatives before optimizing recall and precision, reflecting the higher clinical cost of failing to identify true responders to immune checkpoint inhibitors.

Importantly, the percentage of applicability is not intended to predict treatment response or to serve as a surrogate for predictive accuracy. Rather, it provides a conservative indicator of potential unreliability when uploaded data substantially deviate from the training cohorts. Individual-level predictions remain available for single patients, while applicability offers complementary guidance when cohort-level interpretation is appropriate. Together, these components support cautious and transparent use of transcriptomic predictors in clinical and translational research.

The empirical thresholds for the percentage of applicability were derived from four independent cohorts; additional cohorts will be required to further refine and validate these thresholds. Future work will also examine how the choice of distance metric for quantifying similarity between new samples and responder/non-responder groups influences applicability estimates. Finally, we plan to extend the GUI to include a response predictor for melanoma, which has relatively high objective response rates (∼40–45%) to immune checkpoint inhibitors and represents a clinically important population for such tools.

## Conclusions

In summary, we present the ImmunoResponse Predictor, a user-friendly GUI built on LogitDA and identified gene signatures for mUC and mRCC. By combining clinically informed score cutoffs with an explicit assessment of cohort-level applicability, the tool enables cautious interpretation of immunotherapy response predictions under potential distributional shift. Designed to support rather than replace clinical judgment, the GUI provides individual-level predictions together with safeguards highlighting limitations when new data fall outside the validated domain. This framework represents a practical step toward responsible translation of transcriptomic predictors into clinical and translational research, and its public web-based deployment enables access from any location with an internet connection.

##  Supplemental Information

10.7717/peerj.21553/supp-1 Supplemental Information 1 The output and annotations of LogitDA

10.7717/peerj.21553/supp-2 Supplemental Information 2 Our signature for mUC/mRCC outperformed the other well-known ones

10.7717/peerj.21553/supp-3 Supplemental Information 3 Output of the ImmuneResponse predictor for ORR information-based cosine distance

10.7717/peerj.21553/supp-4 Supplemental Information 4 Single sample output PCD(mUC)

10.7717/peerj.21553/supp-5 Supplemental Information 5 Architecture diagram of ImmunoResponse Predictor

10.7717/peerj.21553/supp-6 Supplemental Information 6 Sample test file for mUC

10.7717/peerj.21553/supp-7 Supplemental Information 7 Sample test file for mRCC

10.7717/peerj.21553/supp-8 Supplemental Information 8 Technical report of mUC/mRCC

## Additional Information and Declarations

### Data Availability

The following information was supplied regarding data availability: The data and test files for the mUC and mRCC models are available in the Supplemental Files. The code is available at GitHub and Zenodo: - https://github.com/gshieh/ImmunoResponsePredictor
- gshieh. (2026). gshieh/ImmunoResponsePredictor: ImmunoResponsePredictor (v1.0.1). Zenodo. https://doi.org/10.5281/zenodo.18616476
The data is available at: http://research-pub.gene.com/IMvigor210CoreBiologies/
The raw clinical data of PCD4989g are available at GitHub: https://github.com/EGA-archive/ega-download-client
Data for the clinical trial IMvigor210 is available in European Genome-Phenome Archive (EGA) under accession number EGAS00001002556. The raw RNA-seq and clinical data for IMmotion150 and PCD4989g are third-party controlled-access clinical trial data and cannot be redistributed by the authors because access is governed by Genentech/EGA data-use agreements. Qualified researchers may request access through the original data providers/repositories, where applicable. To enable reproducibility of the analyses reported in this article, the processed gene-expression matrices used by the ImmunoResponse Predictor, response labels used for model training/testing, gene signatures, analysis scripts, and example input/output files are available at the following links: - https://zenodo.org/records/18616476
- https://github.com/gshieh/ImmunoResponsePredictor. These processed data are sufficient to reproduce the reported LogitDA scores, classifications, validation metrics, and applicability analyses.
