# Supplementary material for: ImmunoResponse Predictor: a GUI for accurate response prediction to immunotherapy
Source: PeerJ. 2026 Aug 6;14:e21553. doi: 10.7717/peerj.21553 (PMC13453136; doi:10.7717/peerj.21553)
Supplement: Supplementary file 1 [file peerj-14-21553-s008.pdf]

Gene signature for response prediction to immunotherapy in mUC and RCC

Peter Langfelder[1, 2, 3], En-Tni Lin[1], Yi-Ta Tsai[4], Tai-Lung Cha[5], Grace S. Shieh[1, 6, 7, 8*]

[1] Institute of Statistical Science, Academia Sinica, Taipei, Taiwan

[2] Center for Neurobehavioral Genetics, The Jane and Terry Semel Institute for Neuroscience and Human Behavior, University of California, Los Angeles, Los Angeles, United States

[3] Department of Psychiatry and Biobehavioral Sciences, David Geffen School of Medicine, University of California, Los Angeles, Los Angeles, United States

[4] Graduate Institute of Life Sciences, National Defense Medical Center, Taipei, Taiwan

[5] National Institute of Cancer Research, National Health Research Institutes, Miaoli, Taiwan

[6] Bioinformatics Program, Taiwan International Graduate Program, Academia Sinica, Taipei, Taiwan

[7] Data Science Degree Program, Academia Sinica and National Taiwan University, Taipei, Taiwan

[8] Genome and Systems Biology Degree Program, Academia Sinica and National Taiwan University, Taipei, Taiwan

***Correspondence:**

Grace S. Shieh

gshieh@stat.sinica.edu.tw

Abstract

To date, immune checkpoint inhibitors (ICIs) have emerged as a leading treatment for metastatic cancer, significantly improving patient survival while causing relatively few side effects. However, the objective response rate for ICIs remains low—approximately 20-25% in urothelial carcinoma (UC) and renal cell carcinoma (RCC), underscoring the urgent need for predictive response biomarkers. Several state-of-the-art signatures have

been revealed in top-tier journals, highlighting the importance of this field. As the number of genes (~20,000) far exceeds the sample sizes of typical training sets (generally ≤ 300), we first developed feature selection procedures to reduce the number of features to a few hundred. We then trained multiple machine learning classifiers using the selected genes and the IMvigor210 and IMmotion150 datasets, which includes RNA-seq and clinical data from ~298 and 77 patients with metastatic UC (mUC) and metastatic RCC (mRCC), respectively. Notably, our predictor LogitDA, using the identified 49 (27)-gene signature, achieved a prediction AUC of 0.75 (0.72) in independent datasets, PCD4989g (mUC and mRCC). Moreover, our signature outperformed six state-of-the-art signatures, PD-L1 IHC, and five tumor microenvironment signatures, including IFN-γ, T-effector, and T-cell exhaustion signatures. Our signature for mRCC was second to T exhaust in prediction AUC but surpassed the six known signatures in prediction accuracy. Finally, from our signature, we identified key prognostic biomarkers, with the top five markers *LYRM1*, *RFC4*, *CENPL*, *SPAG5*, and *CACYBP* (Benjamini-Hochberg adjusted $P < 0.0025$) in the IMvigor210 cohort, and *FLNC* and *NIPAL1 (*BH-adjusted $P < 0.02$) in the IMmotion150 cohort..

Keywords: biomarker, cancer, immunotherapy, machine learning, regression, prediction

Introduction

Almost 90% of human cancer deaths are due to metastases. Currently, the bottleneck in cancer treatment lies in metastasis. For several types of metastatic cancers, immune checkpoint inhibitors (ICIs) have improved the clinical treatment of patients. For example, the PD-L1 inhibitor atezolizumab can treat patients with metastatic urothelial carcinoma (mUC) (Ayers et al., 2017; Mariathasan et al., 2018; Nassar et al., 2019). Programmed cell death 1 (PD-1) and programmed cell death-ligand 1 (PD-L1) are proteins that play important roles in the human immune system. PD-L1/PD-1 inhibitors, including atezolizumab, have shown durable responses in patients with previously treated mUC (Chen et al., 2020). Specifically, atezolizumab is a humanized engineered monoclonal antibody that selectively targets PD-L1 to block its interaction with PD-1 and the co-stimulatory molecule B7.1, to reinvigorate tumor-specific T-cell immunity. If patients respond to PD-1 or PD-L1 inhibitor therapy, their overall survival (OS) can be significantly extended. Although ICIs are effective and have few advent effects, the

objective response rate (ORR) of patients is relatively low, only around 20% in urothelial carcinoma and renal cell carcinoma (RCC) (Deleuze et al., 2020; Banchereau et al., 2021), which makes identifying biomarkers to detect responders before drug administration essential but challenging (Havel et al., 2019).

Banchereau and colleagues found that both tumor mutation burden (TMB) and PD-L1 achieved low AUC for the integrated IMvigor210(mUC), POPLAR, and IMmotion150(RCC) dataset, and their result suggested RNA-seq recapitulated the effect of TMB and PD-L1 (Banchereau et al., 2021). They also indicated that a cancer-specific approach might be more promising than a cross-cancer approach on the prediction of response to ICIs. Thus, we took a cancer-specific approach. In this study, we aim to identify gene signatures based on RNA-seq to predict the response of patients with

metastatic UC and RCC to atezolizumab, and to improve the ORR of these patients, which is crucial for overcoming the bottleneck in cancer treatment with immunotherapy.

Data-driven ML methods usually failed to generalize response prediction to new test sets

(Kong et al., 2022). Thus, we tested whether transfer learning can improve feature selection and training of classifiers, compared to purely data-driven methods. As the sample size of ICI-treated patients are limited compared to the number of genes (features) in human, feature selection is critical. Specifically, we incorporated unsupervised domain adaptation (DA) (Ganin and Lempitsky, 2015; Mourragui et al., 2019; Yuan et al. 2023) in the feature selection procedures to reduce ~17K genes to a few hundred.

Next, we trained four classifiers, namely logit, lasso, support vector machine (SVM) and random forest, via cross-validation using RNA-seq and clinical data of IMvigor210 and IMmotion150 for mUC and RCC, respectively. Of the four trained predictors, LogitDA, a logit regression-based model with our feature selection procedures, overall performed the best in the cross-validation and across study experiments of IMvigor-PCD(mUC) and IMmotion-PCD(RCC), where IMvigor-PCD(mUC) denote using IMvigor210 to train a classifier and test the trained predictor on the independent set PCD4989g(mUC). LogitDA also revealed effective gene signature for mUC and RCC, respectively.

We further compared our gene signature for mUC (RCC) to six well-known signatures, which were able to predict the response of cancer patients to ICI treatments well. We considered PD-L1 expression for atezolizumab, and five tumor microenvironment

(TME)-associated signatures, comprising IFN-γ signature (Ayers et al., 2017), T cell dysfunction signature (Jiang et al., 2018), T-effector signature (tGE8; Mariathasan et al., 2018), and T inflamed signature (Cristescu et al., 2018). The IFN-γ signature comprises of 18 IFN-γresponsive genes related to antigen presentation, adaptive immune resistance, and was shown to be associated with clinical response to pembrolizumab across nine tumor types (Ayers et al., 2017). Cristescu and colleagues used >300 patient samples across 22 tumor types to derive T cell-inflamed gene expression profile (GEP), which consisted of 18 genes involved in antigen presentation, chemokine expression, and adaptive immune resistance. Moreover, the eight cytotoxic T cell gene expression (tGE8) signature, composed of *IFNG*, *CXCL9*, *CD8A*, *GZMA*, *GZMB*, *CXCL10*, *PRF1*, and *TBX21*, was demonstrated to be highly correlated with PD-L1 expression in immune cells and was significantly increased in responders, compared to non-responders (Mariathasan et al., 2018; Powles et al., 2019). The Tumor Immune Dysfunction and Exclusion (TIDE) method (Jiang et al., 2018) revealed 50 genes as markers for T cell exhaustion (denoted as T exhaust) from 189 human cancer studies, using survival outcomes as surrogate of response to ICIs and pre-treatment GEPs. The CD8T signature (denoted as CD8T; Lakatos et al., 2020) comprises of *CD8A*, *CD8*B, *GZMA*, *GZMB*, *PRF1* involved in CD8+ T cell.

We found that our 49- and 27-gene signature for mUC and RCC surpassed the aforementioned six known signatures in terms of prediction AUC and accuracy, using IMvigor-PCD(mUC) and IMmotion-PCD(RCC), respectively. We further integrated the six well-known signatures to our signatures, and found that the integrated signature for mUC did not improve the response prediction to ICIs in mUC. Our signature for RCC outperformed the remaining signatures in terms of prediction accuracy. We further shown that using our signature for mUC and tumor mutation burden (TMB), an established genomic biomarker of the ICI response, improved the response prediction to atezolizumab-treated patients (IMvigor210), compared with TMB-alone predictions. Finally, we revealed several prognostic markers for mUC and RCC, respectively, which are able to predict overall survival of patients with mUC and RCC. Taken together, our method (LogitDA) revealed gene signature for mUC and RCC, which helped well-known biomarkers improve response prediction to ICIs.

Materials and Methods

mUC and RCC patient data sets

We have downloaded gene expression data and clinical information of patients with metastatic urothelial carcinoma (mUC) and renal cell carcinoma (RCC), respectively, from Mariathasan et al. (2018) and Banchereau et al. (2021). These datasets consist of the clinical response of 298 patients from a single-arm phase II trial of atezolzumab as first-line treatment in patients with primarily mUC (IMvigor210, NCT02108652; Balar et al., 2017), and 77 RCC patients from a randomized phase II trial of atezolizumab vs. atezolizumab+bevacizumab vs. sunitinib in front line RCC (IMmotion150, NCT01984242; McDermott et al., 2018). These datasets served as training sets.

Further, we requested data of the phase I trial of atezolizumab (PCD4989g, NCT01375842; Herbst et al., 2014) from Genentech (South San Francisco, USA). This data contained whole transcriptome profiles along with clinical information of 94 and 58 patients with mUC and RCC, respectfully, and was used as test data sets. Resources of the raw data are stated in the Data Availability section. Each data set has 30K and more transcripts which have expression values in counts. The details of each training and test dataset, such as the cancer type, the number of responders, and non-responders, and the percentage of responders are provided in Table 1.

RNA-seq data processing and normalization

Whole transcriptomic profiles of IMvigor210 (IMmotion150, and PCD4989g) were downloaded in raw read (FASTQ) format; the reads were aligned and converted to gene counts by kallisto (Bray et al., 2016). After applying DEseq2 to identify differentially expressed (DE) genes, we normalized the counts using $\log_2$ (TPM+1) (Wagner et al., 2012) for each sample of training and test sets.

The clinical responses of IMvigor210, IMmotion150, and PCD4989g, which followed the Response Evaluation Criteria in Solid Tumors version 1.1 (RECIST 1.1; Schwartz et al., 2016), were classified to the complete response (CR), partial response (PR), stable disease (SD), and progressive disease (PD), respectively. Moreover, for each dataset we grouped patients into responders (CR/PR) and non-responders (SD/PD), respectively. Note that out of 348 patients in IMvigor210, only clinical response of 298 patients is

available; these patients are primarily with mUC, in addition to liver, lung and other cancers.

Standardization of training and test data

To reduce the influence of systematic technical differences between training and test data sets, we first applied independent standardization (denoted as ST) to IMvigor210 and PCD4989g(mUC), that scaled each gene to mean 0 and variance 1 separately in training and test data, as the proportions of the two outcomes CR/PR and SD/PD are similar in IMvigor210 and PCD4989g(mUC) (Table 1) which satisfied the assumption of ST. For IMmotion150 and PCD4989g(RCC) whose proportions of responders differed much (Table 1), we first applied ComBat (Johnson et al., 2007) to reduce the batch effects. Next, we applied quantile normalization (QN, Bolstad et al., 2003) to the merged set of IMmotion150 and PCD4989g(RCC) samples. QN equalizes all quantiles of each sample to a common reference "sample" whose quantiles are defined as the average of the corresponding quantiles of all samples in the data set. ST, Combat and QN were only applied to RNA-seq data.

Feature Selection

For feature selection, we have implemented the following procedures. (1) identifying differentially expressed (DE) genes of responders versus non-responders by DEseq2 (Love et al., 2014), excluding non-informative genes (HIST and LOC), (2) computing between-group to within-group sums of squares ratio (BW-ratio; Dudoit et al., 2002) for the DE genes, where the two groups refer to responders and non-responders, and (3) applying unsupervised domain adaptation (DA; Mourragui et al., 2019) to sift genes which pass step (2). The flowchart of the proposed scheme is depicted in Figure 1.

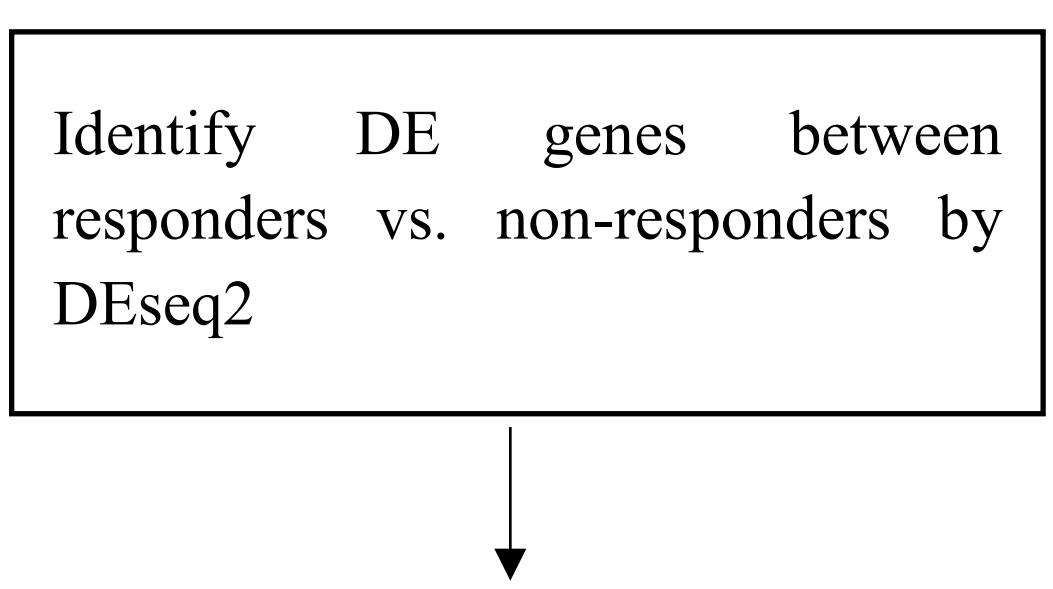

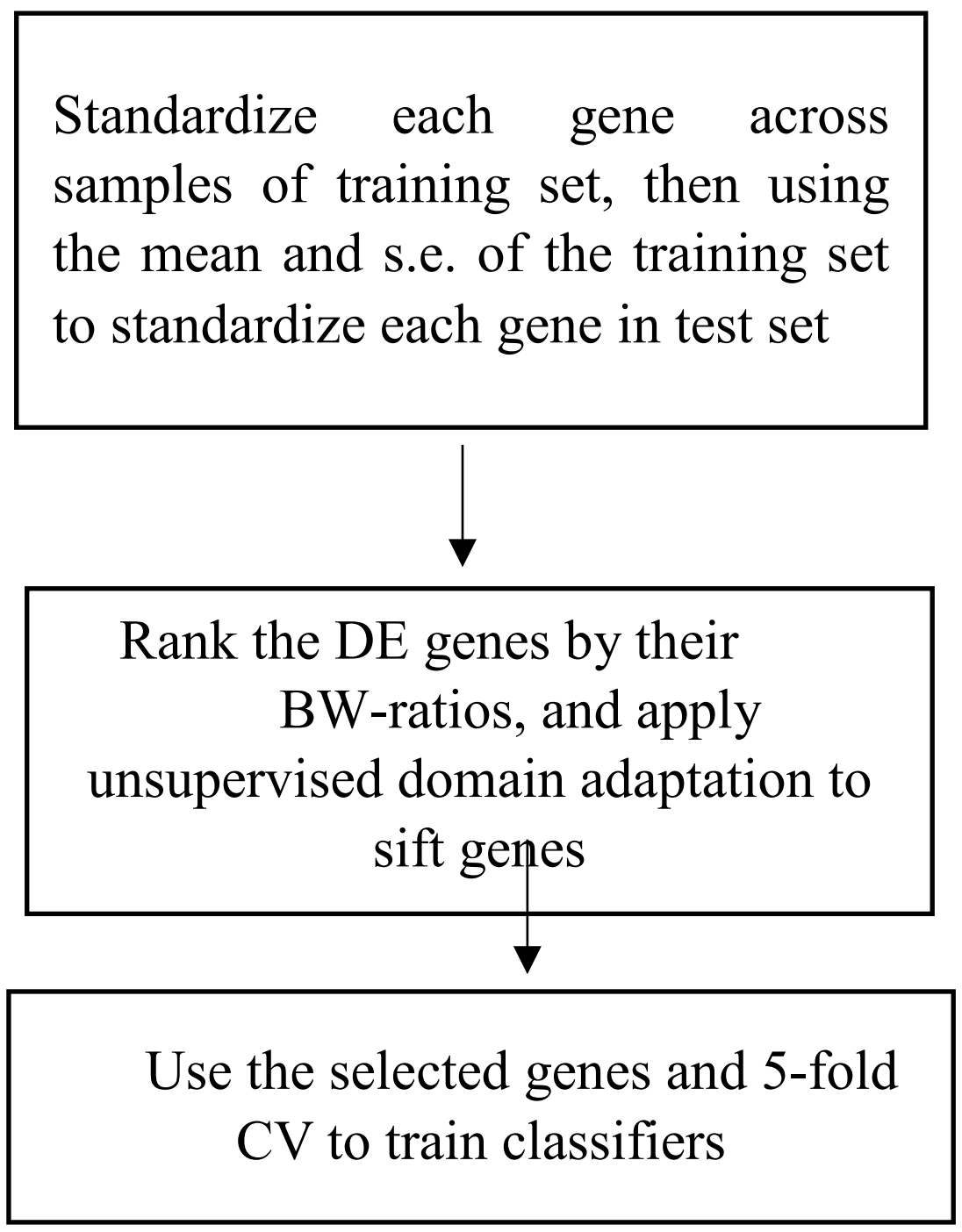

Figure 1. The flowchart of our scheme, where the DE genes of IMvigor210 (IMmotion150) were selected by FDR < 0.10.

Details of the feature selection procedures

(1) To find DE genes between responders vs. non-responders, we used DESeq2 (Love et al., 2014) which assumes the read counts follow negative binomial distributions; this assumption was checked by insignificance from the Kolmogorov and Smirnov test (*P* > 0.10, KS test). For genes that followed negative binomial distributions, we identified 1993 (59) DE genes of IMvigor210 (IMmotion150) (false discovery rate (FDR) < 0.10 (Benjamini and Hochberg, 1995); DEseq2 on responders versus non-responders).

(2) Next, read counts were normalized to $\log_2$(TPM+1), and all DE genes were ranked by their BW-ratios which indicated how well they separate responders from non-responders to a drug (Dudoit et al., 2002; Yuan et al., 2023). The BW-ratio for a gene *j* of subject *i* (x_{ij}) in group *k*, and *k* = 1 (responders) and 2 (non-responders), is defined as follows.

$$\mathrm{BW(j)} = \frac{\sum_{\mathrm{i}} \sum_k I(y_i=k)(\bar{x}_{kj}-\bar{x}_{.j})^2}{\sum_{\mathrm{i}} \sum_k I(y_i=k)(x_{ij}-\bar{x}_{kj})^2},$$

where $\bar{x}_{.j}$ and $\bar{x}_{kj}$ denote the average expression level of gene j across all subjects and across the subjects of group k only, respectively.

The top-p ranked genes are selected, where the parameter p is optimized by grid search with 5-fold CV and 100 repeats. For example, p is evaluated from the union of [15(5)200]) and [210(10)500], where the former denotes the set of numbers from 15 with step size 5 to 200 for IMvigor210; similarly p is evaluated in [15(5)55] for IMmotion150.

(3) Finally, as the training sets (Phase II clinical trial cancer patients) are similar to the test sets (phase I clinical trial cancer patients), unsupervised DA is implemented to ensure that the behavior (probability distribution) of each selected gene (feature), say X_i , across the domains are similar, i.e., $P_S(X_i) \approx P_T(X_i)$, where S and T denote source (training) and target (test) domains, respectively. The similarity of the distributions is measured by the p-values of Kolmogorov-Smirnov (KS) test. Genes with $P \geq \alpha_{\mathrm{DA}}$ are chosen to train the predictor, where α_{DA} in [0.2(0.1)0.8] is optimized by grid search with 5-fold CV and 100 repeats.

Training four classifiers by 5-fold CV and yielding the best predictor LogitDA

After feature selection procedures, we utilized the training set IMvigor210 (IMmotion150) to train four classifiers, namely logistic regression, lasso regression, random forest (RF) and SVM with the RBF kernel (denoted as SVM(RBF)), via 5-fold cross-validation (CV) and 100 repeats to result in the associated predictors.

Specifically, for LogitDA, given a fixed top-ranked p genes, where p is in the union of [15(5)200]) and [210(10)500] for IMvigor210 and α_{DA} in [0.2(0.1)0.8], we trained the hyperparameter λ (the penalty constant of logit ridge regression), p and α_{DA} using 5-fold CV with 100 repeats simultaneously. We used equal spaced grid-search to tune the hyper-parameter p and α_{DA}, and employed a two-stage optimization (Yuan et al., 2023) to determine λ as follows. The scoring function is the average of the area under the receiver operating characteristic

curve (AUC) in all folds. First, let $\lambda = 10^{a_0}$ and $a_0 \in [-3, 0]$ with step size $10^{0.3}$, i.e., we ran 5-fold CV of LogitDA with grid points 10^{-3}, $10^{-2.7}$, … , and 10^{0}, and found the grid point whose associated CV score was the maximum, which was termed 10^{a_1},, e.g., $a_1 = -2.7$. Second, we further evaluated the logit ridge model (LogitDA) with grid points in $[10^{a_1-0.05}, 10^{a_1+0.05}]$ with step size $10^{0.01}$. The grid point $10^{a_{max}}$ whose corresponding CV score is the maximum determines the tuned hyperparameter $\lambda = 10^{a_{max}}$. The logit model with the highest averaged CV AUC determined p and λ, which yielded one LogitDA. Finally, all training data was used to fit LogitDA with the optimized hyperparameters to yield the LogitDA predictor.

For the other classifiers, lasso was optimized similar to LogitDA, while for SVM(RBF) and random forest, we used R packages and the optimization therein.

Statistical analysis and tools

We analyzed data using the DEseq2 and dgof tools in R software to obtain DE genes for responders vs non-responders. Log-rank test was applied to reveal genes which can separate patients into good and poor OS (PFS) for IMvigor210 (IMmotion150). In the remaining data analysis, we used R for logistic ridge regression, support vector machine, random forest, and volcano plot.

Results

LogitDA outperformed three predictors in response prediction of ICI treatments in mUC and RCC

After genes been sifted by our feature selection procedures, we first conducted 5-fold cross-validation (CV) study of LogitDA, lasso, random forest and support vector machine with RBF kernel (SVM(RBF)) on IMvigor210 and IMmotion150, respectively. For IMvigor210, lasso had 2% (3%) higher CV AUC than LogitDA (SVM(RBF)), and random forest performed the worst, where AUC denoted the area under the ROC curve; we refer to Table 2 for details. For IMmotion150, SVM(RBF), LogitDA and lasso performed similarly well, followed by random forest (Table 2).

Let IMvigor-PCD(mUC) denote using IMvigor210 as the training set to train a classifier and test the trained predictor on PCD4989g(mUC), and similarly for IMmotion-PCD(RCC) henceforth. For IMvigor-PCD(mUC), lasso and LogitDA achieved similarly high prediction AUC (0.77 and 0.75), followed by SVM (0.71), and random forest (0.59).

For IMmotion-PCD(RCC), SVM and LogitDA achieved high prediction AUC (0.73 and 0.72, respectively), followed by random forest (0.68), and lasso (0.61). The detailed results comprising prediction AUC, accuracy, true positives (TPs), true negatives (TNs), false positives (FPs), and false negatives (FNs) are in Table 2.

Taken the result of mUC and RCC together, LogitDA outperformed the remaining three predictors in terms of prediction AUC. Next, we compared the performance of our gene signature for response to ICIs to six sets of well-known ones, using IMvigor-PCD(mUC) and IMmotion-PCD(RCC).

INSERT Table 2

Our signature outperformed the six well-known signatures in ICI-treated patients with mUC

In this section, we compare the performance of our signature with the six well-known signatures, which consist of *CD274* mRNA (encoding PD-L1), IFN-γ (Ayers et al., 2017), tGE8 (Mariathasan et al., 2018), T exhaust (Jiang et al., 2018), T inflamed (Cristescu et al., 2018), and CD8T (Lakatos et al., 2020), elaborated in the Introduction section. We first conducted a 5-fold CV study (with 100 repeats) of each signature, using logit regression with our optimization algorithm to result in the corresponding predictors. For the IMvigor210 dataset, our signature achieved a CV AUC of 0.77, followed by the IFN-γ (both equal 0.70), T inflamed (0.69), tGE8 (0.68), T exhaust (0.66) signatures; CD8T and PD-L1 had CV AUC lower than 0.60.

Next, we applied the trained predictors to the independent test set PCD(mUC). Our transcriptomic signature outperformed the six known signatures in terms of a prediction AUC of 0.75, followed by T inflamed (0.70), IFN-γ (0.67), tGE8 (0.65), and the

remainders (0.59 and lower), as demonstrated in Table 3A. The prediction accuracy of our signature for mUC is also the highest (0.71) among all signatures, followed by IFN-γ (0.67) and the remainders (0.64 and lower). As PCD(mUC) does not comprise TMB, we could not test its prediction power. The detailed prediction AUC, accuracy, TPs, TNs, FPs, and FNs are in Table 3A. The scheme of training and test of LogitDA and logit-based predictors with the above signatures, and their associated prediction AUC of PCD(mUC) are displayed in Figure 2A.

Our signature for RCC was second to T exhaust in prediction AUC but surpassed the six known signatures in prediction accuracy

Similarly, a within-study of IMmotion150 (n = 77) cross-validations revealed that our signature achieved an AUC of 0.95, and IFN-γ and T inflamed resulted in CV AUC 0.78 and 0.71, respectively while the remaining ones resulted in 0.62 or lower CV AUC; the trained parameter values and the CV AUC of logit-based predictor with each signature are in Table 3B.

For response prediction of patients with RCC to atezolizumab (PCD(RCC), n = 56), LogitDA with our signature (ours) achieved a prediction AUC of 0.72, which was second to logit regression trained with T exhaust (0.78), but ours outperformed tGE8 (0.65), and the remainders (≤ 0.64). Note that the CV AUC of T exhaust was 0.53, having a serious under-fitting problem. Moreover, the prediction accuracy of ours (0.83) is the highest of all signatures, followed by that of T exhaust (0.69) and the remainders (≤ 0.62); we refer to Table 3B for the detailed prediction result. As PCD4989g did not contain TMB, we could not test its prediction power. The scheme of training and test of LogitDA and logit-based predictors with the above signatures, and their prediction AUC of PCD(RCC) are displayed in Figure 2B.

INSERT Fig. 2

Figure 2. Predictive performance of our signature and six known ones in independent mUC and RCC datasets. (A) The scheme of immunotherapy response predictions in mUC and RCC independent datasets. The datasets and transcriptomic signature used to

train and test the Logit models, and the number of samples in each dataset are shown. (B)-(C) The area under the receiver operating characteristic curve (AUC) for the (B) PCD(mUC), and (C) PCD(RCC) are displayed. The random expectation (AUC = 0.5) is shown in dotted lines.

Ablation study

In this section, we evaluated the contribution of DA. We trained logit regression models with features selected by the first two procedures, using IMvigor210 and IMmotion150 with 5-fold CV to train all parameters except α_{DA}. The trained predictor (denoted as LogitDA-DA) for mUC and RCC comprised 150 and 55 genes, respectively. The corresponding predictors resulted in prediction AUC 0.63 and 0.725 for IMvigor-PCD(mUC) and IMvigor-PCD(RCC), respectively. DA increased prediction AUC 12% for IMvigor-PCD(mUC). For IMmotion-PCD(RCC), LogitDA-DA only increased prediction AUC 0.0075 compared to LogitDA, but it fitted 28 more genes. We further restricted the number of fitted genes to 30 or less, LogitDA-DA for RCC resulted in a prediction AUC of 0.66, thus DA increased 6% of prediction AUC.

Prognostic biomarkers of OS/ PFS revealed for mUC and RCC

To uncover prognostic markers for overall survival (OS) and progression free survival (PFS), we conducted log-rank test for the 49 (27) fitted genes in the predictor for IMvigor210-PCD(mUC) (IMmotion-PCD(RCC)), respectively. For IMvigor-PCD(mUC), we revealed 18 biomarkers with BH-adjusted P (FDR) < 0.01; the top-five ranked biomarkers are *LYRM1*, *RFC4*, *CENPL*, *SPAG5*, and *CACYBP* (all with BH-adjusted P < 0.0025). The Kaplan-Meier plots of these biomarkers are in Figure 3 (A-E). Of the genes in the predictor of IMmotion-PCD(RCC), we uncovered two prognostic biomarkers for PFS with BH-adjusted P < 0.02 (log-rank test), namely *FLNC* and *NIPAL1* (BH-adjusted P = 0.017 and 0.018, respectively). The KM-curves of *FLNC* and *NIPAL1* are shown in Figure 3 (F-G).

Figure 3. Prognostic biomarkers for overall survival (OS) of the mUC (IMvigor210) cohort (A-E) and for progression-free survival (PFS) of the RCC (IMmotion150) cohort (F-G), split by transcriptional expression of *LYRM1*, *RFC4*, *CENPL*, *SPAG5*, *CACYBP* (A-E), or *FLNC* and *NIPAL1* (F-G). Gene expression level is defined as high (≥ median) or low (< median) within each cancer.

Discussion

In this study, we developed feature selection procedures and a ML-based predictor (LogitDA). We demonstrated LogitDA robustly predicted response of patients with mUC and RCC to PD-L1 inhibitor (atezolizumab), respectively, and identified effective gene signatures. Next, using LogitDA we showed that the identified signature for mUC, outperformed *CD274* mRNA (encoding PD-L1) and the five well-known TME signatures in terms of prediction AUC and accuracy. For RCC, our signature ranked second to T exhaust in prediction AUC (6% less), but ours comprised 28 fewer genes than T exhaust, and ours surpassed all known signatures studied in prediction accuracy.

Furthermore, we integrated our signature with each of the known six signatures, and demonstrated that our signature for mUC performed the best (with a prediction AUC of 0.75), followed by the remaining integrated signatures. For RCC, the integrated signature ours+INF-γ did improve the prediction AUC (from 0.72 to 0.76). Nevertheless, the prediction accuracy of our 27-gene signature for RCC (0.83) performed equivalently as the top-1 ranked PD-L1+ours (0.84). It is interesting to observe that PD-L1 expression only increased 1% prediction AUC of our signature in RCC. These findings indicate that our method can reveal effective signatures from ICI-treated patients with mUC and RCC, and can help stratify patients with mUC/RCC for response to atezolizumab.

TMB is a well-known genomic biomarker of response to immunotherapy in several cancer types, e.g., melanoma. A higher TMB is likely to increase neoantigen presentation, and subsequently increase T-cell infiltration and ICI treatment efficacy. Unfortunately, both test datasets (PCD(mUC) and PCD(RCC)) we assessed do not comprise TMB levels. Thus, we studied the CV result of TMB alone and TMB+ours using IMvigor210 and IMmotion150. The LOOCV AUC of TMB and TMB+ours for mUC (RCC) are 0.44 and 0.78 (0.34 and 0.97), respectively. Importantly, we found that

the combined signature TMB+ours correctly reclassified 28 non-responders from predicted responders using TMB alone (denoted by R2NR), and correctly reclassified 12 responders from predicted non-responders using TMB alone (NR2R) for mUC. Among the 52 RCC patients with TMB levels (8 true responders), TMB predicted 30 Rs (22 NRs) and the combined signatures TMB+ours correctly reclassified 23 (2) to NRs (Rs), respectively. This result suggests that TMB combined our transcriptomic signature can improve ICI-response prediction in mUC and RCC.

Using cibersort (Newman et al., 2015), we deconvoluted bulk RNA-seq of IMvigor210 and found that plasma cells and macrophages M1 cells were significantly positively associated with response of patients with mUC ($P < 0.05$, FDR < 14%; Welch test). However, we did not find any cell type significantly associated with immune response of patients with RCC, using IMmotion150. We further checked the 49-gene signature for mUC against the reference marker gene expression matrix (LM22.txt of cibersort), and found *MAST1* and *CXCL9* were overlapping genes and their overexpression were positively correlated with response to atezolizumab (IMvigor210).

Interestingly, an overfitting problem occurred when we applied the trained predictors based on Logit, lasso, random forest, and SVM to response prediction of patients with RCC (IMmotion-PCD(RCC)), in which the prediction AUC dropped 21%-33% from the corresponding CV AUC. This may be because IMmotion150 and PCD(RCC) are more heterogeneous than IMvigor210 and PCD(mUC), while the optimization aimed mainly on CV AUC of training sets. Thus, a future research direction is to investigate a measure for the heterogeneity (or “distance”) between training and test data, and tighten the criterion of domain adaptation accordingly. Another direction of future research is to study data augmentation methods which may circumvent the overfitting problem of IMmotion-PCD(RCC). Finally, we shall study integration of single cell RNA-seq with bulk RNA-seq data of mUC/RCC; this may elucidate which cell type proportions, and which genes in which cell types are involved in response to ICIs such as atezolizumab.

Data availability

Data for the clinical trial IMvigor210 is available in European Genome-Phenome Archive (EGA) with the reference number of EGAS00001002556. Also, it is included in the data package IMvigor210CoreBiologies for the R statistical computing environment, available at http://research-pub.gene.com/IMvigor210CoreBiologies/. Data for the

clinical trials IMmotion150 and PCD4989g are available under restricted access in European Genome-Phenome Archive with the reference number EGAS00001004343. We request raw data and clinical data access from Genentech's internal stranded pipeline (South San Francisco, USA) and downloaded data using pyEGA3 (https://github.com/EGA-archive/ega-download-client).

References

1. Ayers M, Lunceford J, Nebozhyn M, Murphy E, Loboda A, Kaufman DR, et al. IFN-γ–related mRNA profile predicts clinical response to PD-1 blockade. *The Journal of clinical investigation*. (2017) 127:2930-40. doi:10.1172/JCI91190

2. Mariathasan S, Turley SJ, Nickles D, Castiglioni A, Yuen K, Wang Y, et al. TGFβ attenuates tumour response to PD-L1 blockade by contributing to exclusion of T cells. *Nature*. (2018) 554:544-8. doi:10.1038/nature25501

3. Nassar AH, Mouw KW, Jegede O, Shinagare AB, Kim J, Liu C-J, et al. A model combining clinical and genomic factors to predict response to PD-1/PD-L1 blockade in advanced urothelial carcinoma. *British journal of cancer*. (2020) 122:555-63. doi:10.1038/s41416-019-0686-0

4. Chen Z, Zhou L, Liu L, Hou Y, Xiong M, Yang Y, et al. Single-cell RNA sequencing highlights the role of inflammatory cancer-associated fibroblasts in bladder urothelial carcinoma. *Nature communications*. (2020) 11:5077. doi:10.1038/s41467-020-18916-5

5. Deleuze A, Saout J, Dugay F, Peyronnet B, Mathieu R, Verhoest G, et al. Immunotherapy in renal cell carcinoma: the future is now. *International Journal of Molecular Sciences*. (2020) 21:2532. doi:10.3390/ijms21072532

6. Banchereau R, Leng N, Zill O, Sokol E, Liu G, Pavlick D, et al. Molecular determinants of response to PD-L1 blockade across tumor types. *Nature communications*. (2021) 12:3969. doi:10.1038/s41467-021-24112-w

7. Havel JJ, Chowell D, Chan TA. The evolving landscape of biomarkers for checkpoint inhibitor immunotherapy. *Nature Reviews Cancer*. (2019) 19:133-50. doi:10.1038/s41568-019-0116-x

8. Kong J, Ha D, Lee J, Kim I, Park M, Im S-H, et al. Network-based machine learning approach to predict immunotherapy response in cancer patients. *Nature communications*. (2022) 13:3703. doi:10.1038/s41467-022-31535-6

9. Ganin Y, Lempitsky V, editors. Unsupervised domain adaptation by backpropagation. International conference on machine learning; 2015: PMLR.

10. Mourragui S, Loog M, Van De Wiel MA, Reinders MJ, Wessels LF. PRECISE: a domain adaptation approach to transfer predictors of drug response from pre-clinical models to tumors. *Bioinformatics*. (2019) 35:i510-i9. doi:10.1093/bioinformatics/btz372

11. Yuan S, Chen Y-C, Tsai C-H, Chen H-W, Shieh GS. Feature selection translates drug response predictors from cell lines to patients. *Frontiers in Genetics*. (2023) 14:1217414. doi:10.3389/fgene.2023.1217414

12. Jiang P, Gu S, Pan D, Fu J, Sahu A, Hu X, et al. Signatures of T cell dysfunction and exclusion predict cancer immunotherapy response. *Nature medicine*. (2018) 24:1550-8. doi:10.1038/s41591-018-0136-1

13. Cristescu R, Mogg R, Ayers M, Albright A, Murphy E, Yearley J, et al. Pan-tumor genomic biomarkers for PD-1 checkpoint blockade–based immunotherapy. *Science*. (2018) 362:eaar3593. doi:10.1126/science.aar3593

14. Powles T, Kockx M, Rodriguez-Vida A, Duran I, Crabb SJ, Van Der Heijden MS, et al. Clinical efficacy and biomarker analysis of neoadjuvant atezolizumab in operable urothelial carcinoma in the ABACUS trial. *Nature medicine*. (2019) 25:1706-14. doi:10.1038/s41591-019-0628-7

15. Lakatos E, Williams MJ, Schenck RO, Cross WC, Househam J, Zapata L, et al. Evolutionary dynamics of neoantigens in growing tumors. *Nature genetics*. (2020) 52:1057-66. doi:10.1038/s41588-020-0687-1

16. Balar AV, Galsky MD, Rosenberg JE, Powles T, Petrylak DP, Bellmunt J, et al. Atezolizumab as first-line treatment in cisplatin-ineligible patients with locally advanced and metastatic urothelial carcinoma: a single-arm, multicentre, phase 2 trial. *Lancet*. (2017) 389:67-76. doi:10.1016/s0140-6736(16)32455-2

17. McDermott DF, Huseni MA, Atkins MB, Motzer RJ, Rini BI, Escudier B, et al. Clinical activity and molecular correlates of response to atezolizumab alone or in

combination with bevacizumab versus sunitinib in renal cell carcinoma. *Nature medicine*. (2018) 24:749-57. doi:10.1038/s41591-018-0053-3

18. Herbst RS, Soria J-C, Kowanetz M, Fine GD, Hamid O, Gordon MS, et al. Predictive correlates of response to the anti-PD-L1 antibody MPDL3280A in cancer patients. *Nature*. (2014) 515:563-7. doi:10.1038/nature14011

19. Schwartz LH, Seymour L, Litière S, Ford R, Gwyther S, Mandrekar S, et al. RECIST 1.1–Standardisation and disease-specific adaptations: Perspectives from the RECIST Working Group. *European journal of cancer*. (2016) 62:138-45. doi:10.1016/j.ejca.2016.03.082

20. Bray NL, Pimentel H, Melsted P, Pachter L. Near-optimal probabilistic RNA-seq quantification. *Nature biotechnology*. (2016) 34:525-7. doi:10.1038/nbt.3519

21. Wagner GP, Kin K, Lynch VJ. Measurement of mRNA abundance using RNA-seq data: RPKM measure is inconsistent among samples. *Theory in biosciences*. (2012) 131:281-5. doi:10.1007/s12064-012-0162-3

22. Love M, Anders S, Huber W. Differential analysis of count data–the DESeq2 package. *Genome Biol*. (2014) 15:10-1186. doi:10.1186/s13059-014-0550-8

23. Dudoit S, Fridlyand J, Speed TP. Comparison of discrimination methods for the classification of tumors using gene expression data. *Journal of the American statistical association*. (2002) 97:77-87. doi:10.1198/016214502753479248

24. Benjamini Y, Hochberg Y. Controlling the false discovery rate: a practical and powerful approach to multiple testing. *Journal of the Royal statistical society: series B (Methodological)*. (1995) 57:289-300. doi:10.1111/j.2517-6161.1995.tb02031.x

25. Yin Z, Yan X, Wang Q, Deng Z, Tang K, Cao Z, et al. Detecting prognosis risk biomarkers for colon cancer through multi-omics-based prognostic analysis and target regulation simulation modeling. *Frontiers in Genetics*. (2020) 11:524. doi:10.3389/fgene.2020.00524

26. Zhang B, Vogelzang A, Miyajima M, Sugiura Y, Wu Y, Chamoto K, et al. B cell-derived GABA elicits IL-10+ macrophages to limit anti-tumour immunity. *Nature*. (2021) 599:471-6. doi:10.1038/s41586-021-04082-1

27. Huang D, Wang Y, Thompson JW, Yin T, Alexander PB, Qin D, et al. Cancer-cell-derived GABA promotes β-catenin-mediated tumour growth and immunosuppression. *Nature cell biology*. (2022) 24:230-41. doi:10.1038/s41556-021-00820-9

28. Liang Y-k, Deng Z-k-, Chen M-t, Qiu S-q, Xiao Y-s, Qi Y-z, et al. CXCL9 is a potential biomarker of immune infiltration associated with favorable prognosis in ER-negative breast cancer. *Frontiers in Oncology*. (2021) 11:710286. doi:10.3389/fonc.2021.710286

29. House IG, Savas P, Lai J, Chen AX, Oliver AJ, Teo ZL, et al. Macrophage-derived CXCL9 and CXCL10 are required for antitumor immune responses following immune checkpoint blockade. *Clinical Cancer Research*. (2020) 26:487-504. doi:10.1158/1078-0432.CCR-19-1868

30. Zheng B, Wang S, Yuan X, Zhang J, Shen Z, Ge C. NUF2 is correlated with a poor prognosis and immune infiltration in clear cell renal cell carcinoma. *BMC urology*. (2023) 23:82. doi:10.1186/s12894-023-01258-x

31. Wang L, Shi J, Huang Y, Liu S, Zhang J, Ding H, et al. A six-gene prognostic model predicts overall survival in bladder cancer patients. *Cancer cell international*. (2019) 19:1-15. doi:10.1186/s12935-019-0950-7

32. Newman AM, Liu CL, Green MR, Gentles AJ, Feng W, Xu Y, et al. Robust enumeration of cell subsets from tissue expression profiles. *Nature methods*. (2015) 12:453-7. doi:10.1038/nmeth.3337

Table 1. Overview of the training and test data.

Training set	No. of patients	R	NR	% of Rs	Test set	No. of patients
IMvigor210 (mUC)	298	68	230	22.8	*PCD4989g* (mUC)	94
IMmotion150 (RCC)	77	15	62	19.5	*PCD4989g* (RCC)	58

Table 2. Performances of four classifiers applied to mUC and RCC.

Training-test set	Method	Parameter tuning		Cross validation result		Prediction result					
		p[a]<br>nGenes[a]	α_{DA}	λ[b]	AUC (SE)	AUC (SE)	accuracy	TP	TN	FP	FN
IMvigor-PCD(mUC)	Logistic Ridge Regression	49<br>**150**	0.2	0.14	0.77 (0.00[c])	0.75	0.71	12	55	17	10
IMmotion-PCD(RCC)	Logistic Ridge Regression	27<br>**50**	0.2	0.05	0.95 (0.00[c])	0.72	0.83	4	44	6	4
IMvigor-PCD(mUC)	Lasso Regression	48<br>**145**	0.2	0.03	0.79 (0.01)	0.77	0.70	12	54	18	10
IMmotion-PCD(RCC)	Lasso Regression	17<br>**30**	0.2	0.01	0.95 (0.02)	0.58	0.71	2	39	11	6
IMvigor-PCD(mUC)	Random Forest	65<br>**260**	0.3	No. tree =1000	0.70 (0.01)	0.59	0.69	3	62	10	19
IMmotion-PCD(RCC)	Random Forest	14<br>**25**	0.2	No. tree =1000	0.87 (0.01)	0.66	0.84	1	46	4	7
IMvigor-PCD(mUC)	SVM radial	49<br>**155**	0.2	C=1.33<br>γ =0.004	0.76 (0.01)	0.71	0.74	0	70	2	22
IMmotion-PCD(RCC)	SVM radial	18<br>**35**	0.2	C=0.72<br>γ =0.01	0.96 (0.01)	0.73	0.83	0	48	2	8

[a]p denotes the top-p genes sifted by the feature selection procedure, and nGenes denotes the number of genes passed the first two feature selection procedures.

[b]λ is the penalty constant of logistic ridge and lasso regression

[c]SE equals to "0.00" after rounded to the 3rd digit.

Table 3A. Our signature for mUC outperformed the six known ones using IMvigor210-PCD(mUC)

Signatures	Parameter	CV result	Prediction result						
	λ^a	AUC (SE)	AUC	accurac y	F1-score	TPs	TNs	FPs	FNs
Ours (49)[b]	0.14	0.77 (0.00)	0.75	0.71	0.47	12	55	17	10
PD-L1[c]	1.07	0.57 (0.001)	0.55	0.40	0.40	15	15	42	3
tGE8 (8)	0.05	0.68 (0.01)	0.65	0.61	0.39	12	45	27	10
IFN- γ [d](18)	0.05	0.70 (0.01)	0.67	0.67	0.46	13	50	22	9
T inflamed[d] (17)	0.04	0.69 (0.02)	0.70	0.64	0.43	13	47	25	9
CD8T (5)	1.04	0.58 (0.01)	0.59	0.61	0.39	12	45	27	10
T exhaust (50)	0.15	0.62 (0.02)	0.56	0.55	0.28	8	44	28	14

[a] λ is the penalty constant of logistic ridge regression.
[b] LogitDA with the optimized α_{DA} = 0.2 and λ= 0. 15 resulted in the 49-gene model
[c] After excluding samples with PD-L1 (IHC) missing, the sample size of IMvigor210 and PCD(mUC) were 297 and 75, respectively.
[d] In the IFN- γ and T inflamed signatures, HLA-E was missing in IMvigor-PCD(mUC).

Table 3B. Our signature for RCC outperformed the other well-known ones using IMmotion150-PCD(RCC)

Signature (No. of genes)	Parameter	CV result	Prediction result						
	λ[a]	AUC (SE)	AUC	accuracy	F1-score	TPs	TNs	FPs	FNs
Ours (27)[b]	0.05	0.95 (0.00[c])	0.72	0.83	0.44	4	44	6	4
PD-L1[d]	1.00	0.57 (0.02)	0.53	0.38	0.26	6	15	33	2
tGE8 (8)	0.11	0.60 (0.05)	0.65	0.50	0.26	5	24	26	3
IFN-γ (19)	0.01	0.66 (0.05)	0.56	0.59	0.20	3	31	19	5
T inflamed (18)	0.001	0.66 (0.05)	0.56	0.62	0.21	3	33	17	5
CD8T (5)	0.03	0.65 (0.04)	0.64	0.57	0.32	6	27	23	2
T exhaust (48)[e]	1.06	0.53 (0.05)	0.78	0.69	0.25	3	37	13	5

[a] λ is the penalty constant of logistic ridge regression

[b] LogitDA with the optimized $\alpha_{DA} = 0.2$ and $\lambda = 0.14$ resulted in the 32-gene model.

[c] Excluding samples without PD-L1(IHC), the sample size of PCD(RCC) = 56.

[d] IMmotion-PCD(RCC) does not comprise *CCL3L3* and *POU6F1*, so only 48 genes in the T exhaust signature.

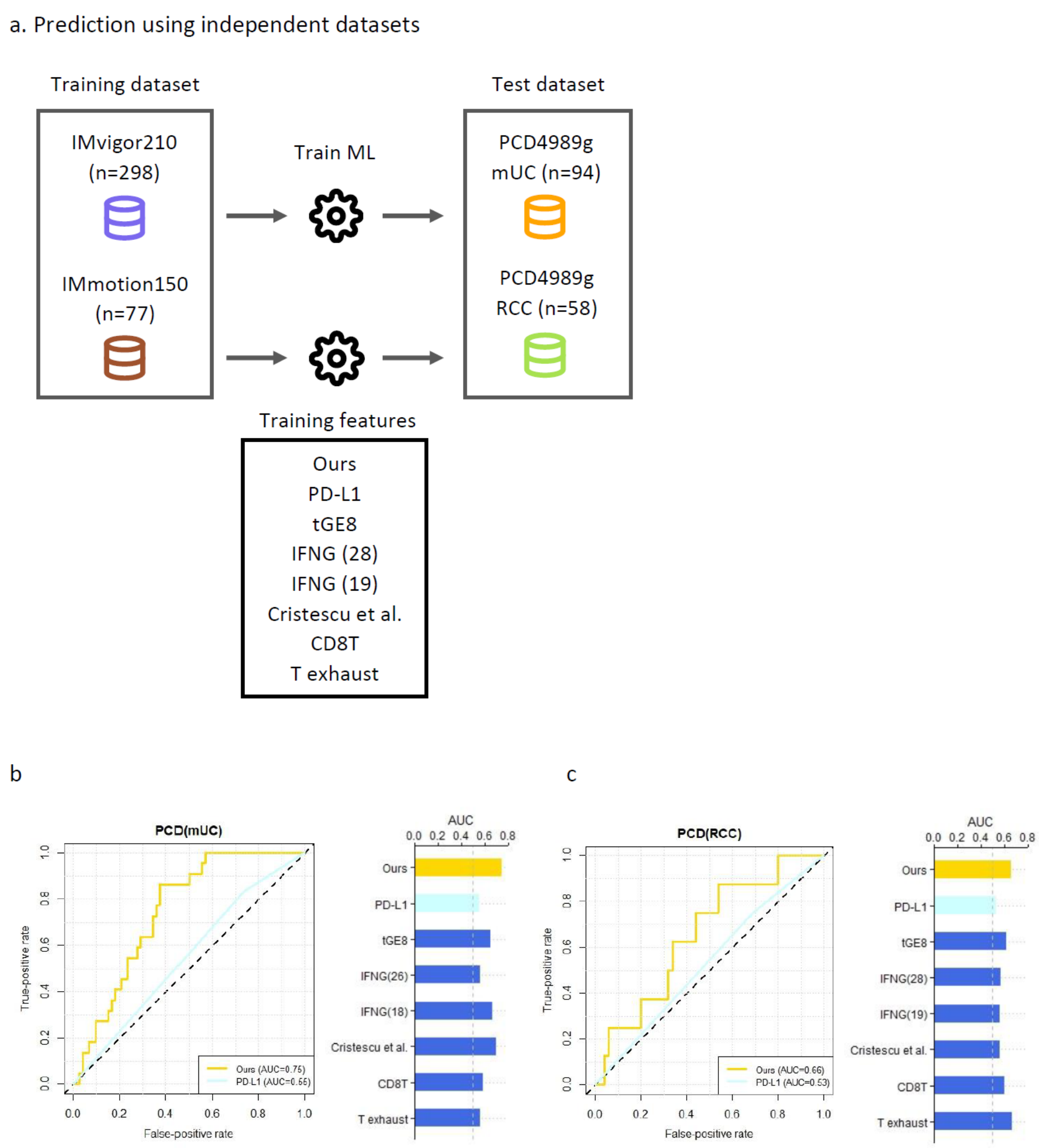

Figure 2. Predictive performance of our signature and six known ones in independent mUC and RCC datasets. (A) The scheme of immunotherapy response predictions in mUC and RCC independent datasets. The datasets and transcriptomic signature used to train and test the Logit models, and the number of samples in each dataset are shown. (B)-(C) The area under the receiver operating characteristic curve (AUC) for the (B)

PCD(mUC), and (C) PCD(RCC) are displayed. The random expectation (AUC = 0.5) is shown in dotted lines.

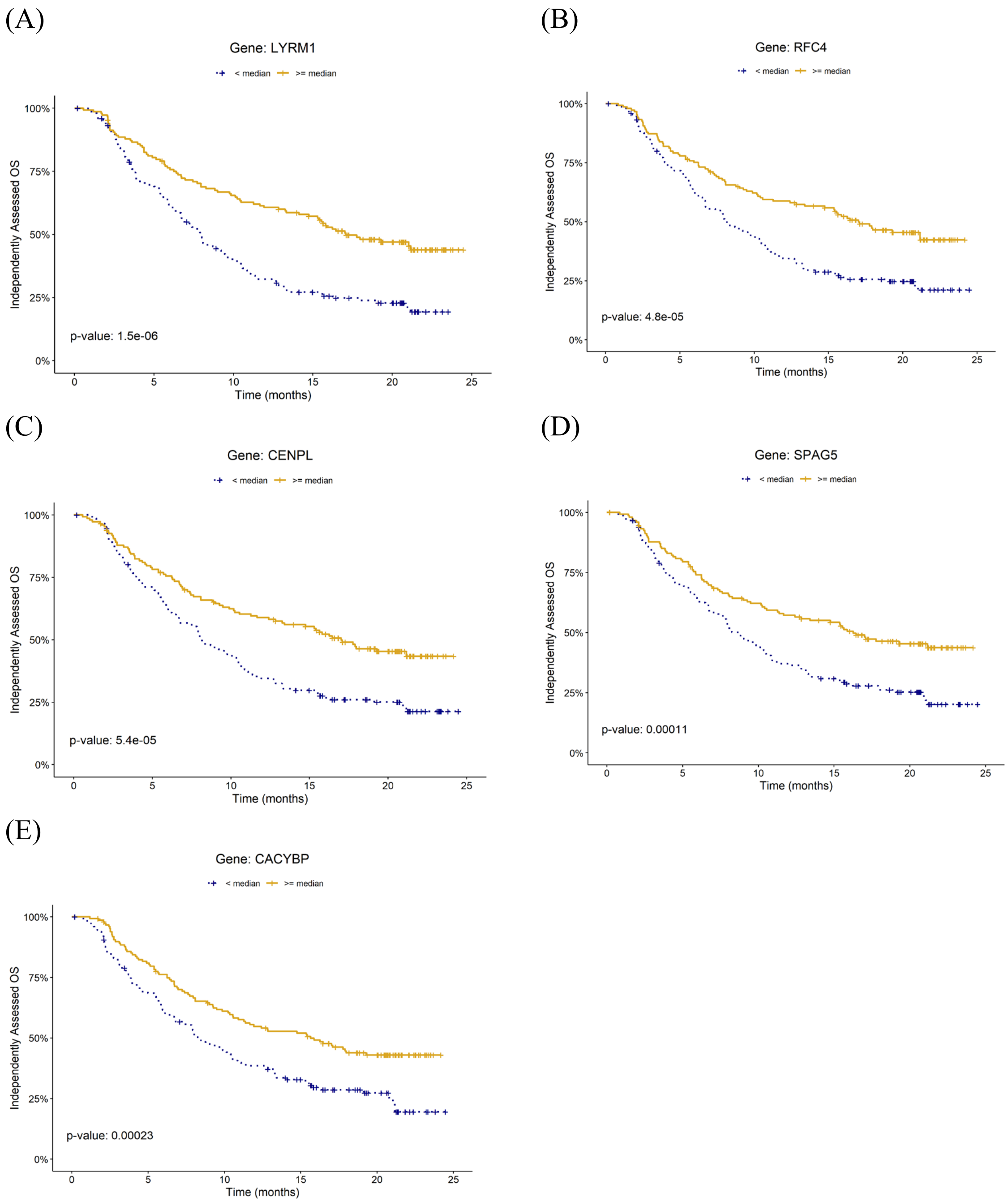

(F)

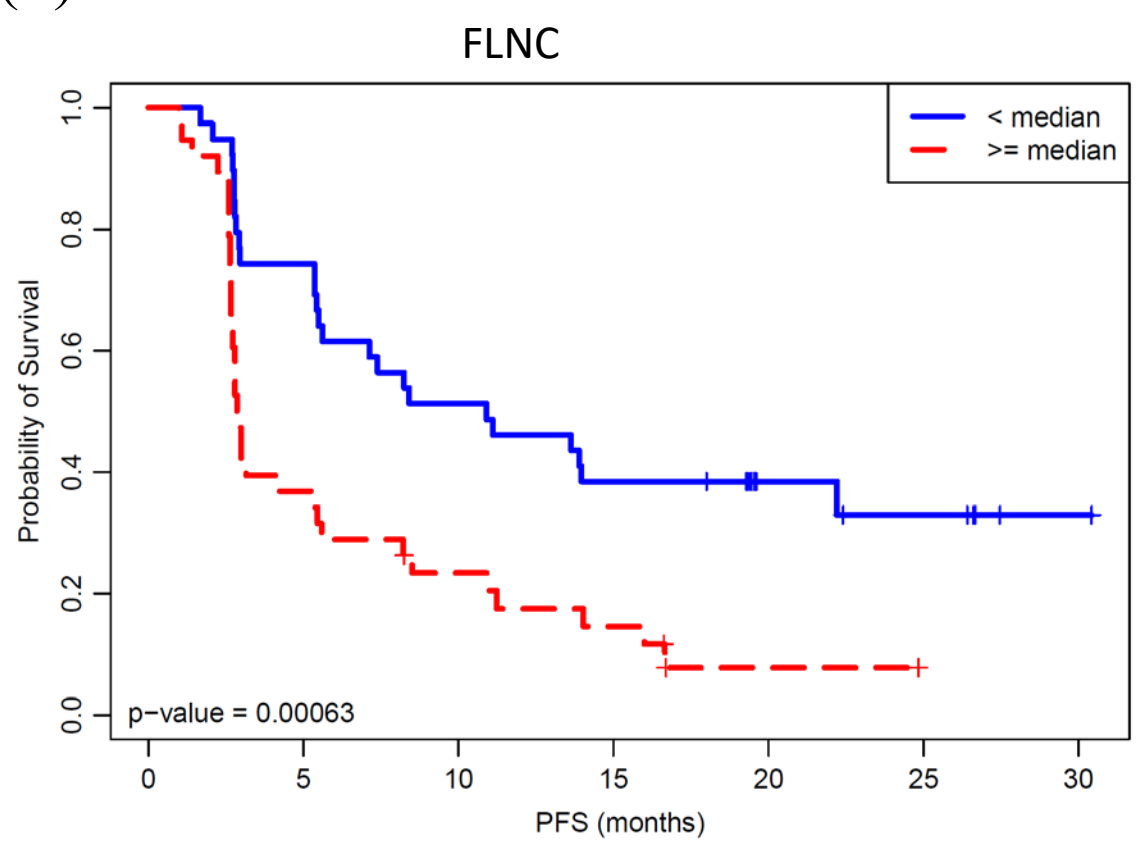

(G)

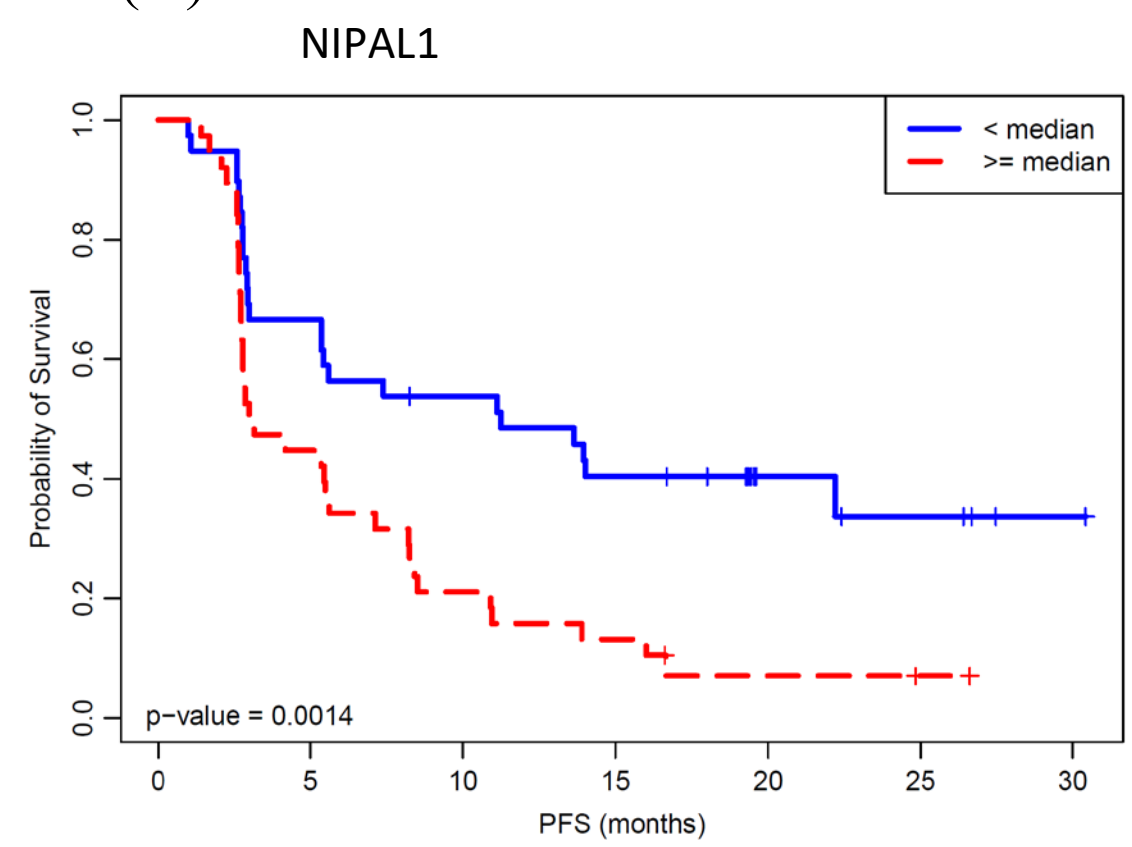

Figure 3. Prognostic biomarkers for overall survival (OS) of the mUC (IMvigor210) cohort (A-E) and for progression-free survival (PFS) of the RCC (IMmotion150) cohort (F-G), split by transcriptional expression of *LYRM1*, *RFC4*, *CENPL*, *SPAG5*, *CACYBP* (A-E), or *FLNC* and *NIPAL1* (F-G). Gene expression level is defined as high (≥ median) or low (< median) within each cancer.
