# Supplementary material for: ImmunoResponse Predictor: a GUI for accurate response prediction to immunotherapy
Source: PeerJ. 2026 Aug 6;14:e21553. doi: 10.7717/peerj.21553 (PMC13453136; doi:10.7717/peerj.21553)
Supplement: Supplementary file 2 [file peerj-14-21553-s002.docx]

These tables were extracted from the technical report:

Langfelder P, Lin E-T, Tsai Y-T, Cha T-L, & Shieh G S. 2025. Gene signature for response prediction to immunotherapy in mUC and RCC. (Tech. Rep. No. 2025-02). Institute of Statistical Science, Academia Sinica.

**Table S2A. LogitDA with our signature outperformed the six existing signatures using IMvigor210-PCD(mUC)**

| **Signatures** | **Parameter** | **CV**  **result** | **Prediction result** | | | | | | |
| --- | --- | --- | --- | --- | --- | --- | --- | --- | --- |
|  | ***λ*^a^** | **AUC**  **(SE)** | **AUC** | **accuracy** | **FNR** | **TPs** | **TNs** | **FPs** | **FNs** |
| Ours (49)^b^ | 0.14 | 0.77  (0.00) | 0.75 | 0.71 | 0.45 | 12 | 55 | 17 | 10 |
| PD-L1^c^ | 1.07 | 0.57  (0.001) | 0.55 | 0.40 | 0.17 | 15 | 15 | 42 | 3 |
| tGE8 (8) | 0.05 | 0.68  (0.01) | 0.65 | 0.61 | 0.45 | 12 | 45 | 27 | 10 |
| IFN-γ^d^(18) | 0.05 | 0.70  (0.01) | 0.67 | 0.67 | 0.41 | 13 | 50 | 22 | 9 |
| T inflamed^d^ (17) | 0.04 | 0.69 (0.02) | 0.70 | 0.64 | 0.41 | 13 | 47 | 25 | 9 |
| CD8T (5) | 1.04 | 0.58  (0.01) | 0.59 | 0.61 | 0.45 | 12 | 45 | 27 | 10 |
| T exhaust (50) | 0.15 | 0.62  (0.02) | 0.56 | 0.55 | 0.64 | 8 | 44 | 28 | 14 |

*^a^*$\lambda$ is the penalty constant of logistic ridge regression.

^b^LogitDA with the optimized α_DA_ = 0.2 and *λ=* 0. 15 resulted in the 49-gene model

^c^After excluding samples with PD-L1 (IHC) missing, the sample size of IMvigor210 and PCD (mUC) were 297 and 75, respectively.

^d^In the IFN-γ and T inflamed signatures, HLA-E was missing in IMvigor-PCD(mUC).

**Table S2B. LogitDA with our signature in mRCC outperformed the six existing ones using IMmotion150-PCD(mRCC)**

| **Signature**  **(No. of genes)** | **Parameter** | **CV**  **result** | **Prediction result** | | | | | | |
| --- | --- | --- | --- | --- | --- | --- | --- | --- | --- |
|  | ***λ*^a^** | **AUC**  **(SE)** | **AUC** | **accuracy** | **FNR** | **TPs** | **TNs** | **FPs** | **FNs** |
| Ours (27)^b^ | 0.05 | 0.95  (0.00^c^) | 0.72 | 0.83 | 0.50 | 4 | 44 | 6 | 4 |
| PD-L1^d^ | 1.00 | 0.57  (0.02) | 0.53 | 0.38 | 0.25 | 6 | 15 | 33 | 2 |
| tGE8 (8) | 0.11 | 0.60  (0.05) | 0.65 | 0.50 | 0.38 | 5 | 24 | 26 | 3 |
| IFN-γ (19) | 0.01 | 0.66  (0.05) | 0.56 | 0.59 | 0.63 | 3 | 31 | 19 | 5 |
| T inflamed (18) | 0.001 | 0.66  (0.05) | 0.56 | 0.62 | 0.63 | 3 | 33 | 17 | 5 |
| CD8T (5) | 0.03 | 0.65  (0.04) | 0.64 | 0.57 | 0.25 | 6 | 27 | 23 | 2 |
| T exhaust (48)^e^ | 1.06 | 0.53  (0.05) | 0.78 | 0.69 | 0.63 | 3 | 37 | 13 | 5 |

*^a^*$\lambda$ is the penalty constant of logistic ridge regression

^b^LogitDA with the optimized α_DA_ = 0.2 and *λ=* 0.14 resulted in the 32-gene model.

^c^Excluding samples without PD-L1(IHC), the sample size of PCD(mRCC) = 56.

^d^ IMmotion-PCD(mRCC) does not comprise *CCL3L3* and *POU6F1*, so only 48 genes in the T exhaust signature.
