# Supplementary material for: ImmunoResponse Predictor: a GUI for accurate response prediction to immunotherapy
Source: PeerJ. 2026 Aug 6;14:e21553. doi: 10.7717/peerj.21553 (PMC13453136; doi:10.7717/peerj.21553)
Supplement: Supplementary file 3 [file peerj-14-21553-s005.docx]

**Upload a test.csv file**

**Select trained model**

**Generate predictions**

**Download predictions**

**Receiving .csv**

**file**

**Select a file…**

**Browse…**

**mUC**

**model**

**mRCC**

**model**

**Select model**

**Interface Unit**

**Check cancer**

**type ?**

**Retrieve the 49**

**signature genes**

**Make prediction,**

**compute the % of**

**applicability**

**Retrieve the 27**

**signature genes**

**mRCC**

**mUC**

**Generate output**

**Computing Unit**

**Preprocessing**

**the data**

**Figure S1.** The architecture diagram of ImmunoResponse Predictor applied to mUC and mRCC, where Rs and NRs denote Responders and Non-Responders, respectively.
